# Recruitment to clinical trials - the use of social media

**DOI:** 10.1186/1745-6215-16-S2-O77

**Published:** 2015-11-16

**Authors:** Will Storrar, Thomas Brown, Lara Balls, Anoop Chauhan, Carole Fogg

**Affiliations:** 1 Portsmouth Hospitals NHS Trust, Portsmouth, UK; 2 NIHR Research Design Service South Central, Portsmouth, UK

## 

The LASER Trial is an NIHR-HTA funded, multicentre, randomised controlled trial of an allergen intervention, (Temperature-controlled Laminar Airflow,) for patients with severe allergic asthma.

To meet our challenging recruitment targets, we have adopted a number of different social media based recruitment strategies to ensure that the trial delivers on time.

Asthma UK and Allergy UK have advertised the trial on their websites and shared the trial with their social media networks via Facebook and Twitter. Asthma UK alone has 27,000 Twitter followers and 35,000 Facebook ‘likes.’ This has raised the profile of the trial and lead to a significant increase in traffic to the trial website where patients can register their interest in participating.

We set up a social media advertising campaign using paid adverts to target asthma patients living within 50km of our recruiting centres. Adverts appear in Facebook and Twitter feeds inviting people to find out more about the trial, directing them to the trial website. This campaign has reached nearly 50,000 people/month and has resulted in a further increase in patient registrations.

We also employed an online recruitment company, Trialbee (http://www.trialbee.com.) Trialbee advertise the trial on social media platforms inviting patients to visit their website where they are screened and, if eligible, referred on to their nearest recruiting centre for screening.

Our social media recruitment strategies have augmented recruitment alongside more traditional means of recruiting patients from the clinical setting.

Social media has the potential to revolutionise recruitment to clinical trials and is currently an underused resource.

